# Dietary Quality Changes Among Cancer Survivors Compared with Age at Cancer Diagnosis: Using the Korean National Health and Nutrition Examination Surveys (KNHANES 2019–2021)

**DOI:** 10.3390/nu18132172

**Published:** 2026-07-04

**Authors:** Sooah Paik, Hyejin Lee, Hye Yeon Koo, In Young Cho, Woo Kyung Bae

**Affiliations:** 1 Department of Family Medicine, Soonchunhyang University Seoul Hospital, Seoul 04401, Republic of Korea; sooah3535@gmail.com; 2 Department of Family Medicine, Seoul National University Bundang Hospital, Seongnam 13620, Republic of Korea; hjinlee@snubh.org (H.L.); tare4645@gmail.com (H.Y.K.); 3 Department of Family Medicine and Supportive Care Center, Samsung Medical Center, 81 Irwon-ro, Gangnam-gu, Seoul 06351, Republic of Korea; 4 Health Promotion Center, Seoul National University Bundang Hospital, Seongnam 13620, Republic of Korea; 5 Department of Family Medicine, Seoul National University College of Medicine, Seoul 03080, Republic of Korea

**Keywords:** cancer survivors, dietary patterns, Diet Quality Index-International, nutritional behavior

## Abstract

**Background/Objectives**: Dietary habits are important modifiable factors influencing survival among cancer patients. The dietary quality among cancer survivors may differ from those of the general population and may vary according to age at cancer diagnosis. This study aimed to compare dietary quality between cancer survivors and the general population and to examine whether age at diagnosis is associated with dietary quality. **Methods**: This retrospective cross-sectional study used data from 8706 adults aged ≥ 30 years (641 cancer survivors and 8065 controls) from the 2019–2021 Korea National Health and Nutrition Examination Survey. Dietary quality was assessed using the Diet Quality Index-International (DQI-I; range 0–100). Survey-weighted multiple linear regression models were used to compare DQI-I scores between cancer survivors and controls. Subgroup analyses were stratified by age at diagnosis, and quadratic age terms were included to assess nonlinear associations. All analyses accounted for the complex survey design. **Results**: Cancer survivors had significantly higher mean DQI-I scores than controls (69.1 ± 0.4 vs. 66.1 ± 0.2; *p* < 0.001). Among survivors diagnosed before age 50, dietary quality was significantly higher in those currently under 65 years than in controls (mean difference +3.02, 95% CI 1.44–4.60), but notably lower in those aged ≥ 65 years (−3.18, 95% CI −6.16 to −0.20). In contrast, survivors diagnosed at age ≥ 50 consistently showed higher dietary quality than controls across all age groups (+3.76, 95% CI 2.83–4.68). **Conclusions**: While cancer survivors generally exhibit better dietary quality than the general population, this positive trend was not observed among younger-onset survivors in older age groups. These findings suggest that age at cancer diagnosis may be associated with dietary quality and highlight the need for sustained, age-specific nutritional support strategies in cancer survivorship.

## 1. Introduction

Cancer survivors are commonly defined as individuals who have been diagnosed with cancer, from the moment of diagnosis through the remainder of life [1]. Advances in early detection and treatment have led to a growing population of cancer survivors worldwide. In South Korea, the 5-year relative survival rate for all cancers increased from 42.9% in 1993–1995 to 72.9% in 2018–2022, resulting in an estimated 2.59 million cancer survivors as of 2022 [2]. This improvement in survival has shifted the focus toward long-term survivorship care, with increasing attention on modifiable lifestyle behaviors.

Lifestyle behaviors—including dietary habits, physical activity, smoking, alcohol consumption, and sleep—are well-established determinants of cancer prognosis, recurrence, and quality of life [1,3]. Evidence suggests that adherence to healthy behavioral patterns after a cancer diagnosis is associated with improved clinical outcomes, such as reduced risks of recurrence and mortality [4]. For instance, smoking cessation after diagnosis significantly reduces the risk of cancer-related and overall mortality, even among long-term smokers [5]. However, favorable behavioral changes are often difficult to maintain over time. A study of older survivors observed declining physical activity and increased sedentary behavior with longer survivorship duration [6], underscoring the need for ongoing support to sustain healthy habits.

Among various lifestyle factors, dietary habits play a central role in survivorship [3,7]. Maintaining a high-quality diet following a cancer diagnosis is associated with improved clinical outcomes, including reduced mortality and recurrence risk [8]. Despite the existence of dietary guidelines for cancer survivors, adherence remains suboptimal. Prior research has indicated that survivors often report poorer dietary habits than the general population [9] and demonstrate low compliance with dietary recommendations [10]. These findings highlight the necessity for personalized nutritional education and support to promote and sustain healthier eating behaviors among cancer survivors.

Effective dietary interventions must account for individual variability, as nutritional needs and behaviors differ across many subgroups [11]. For cancer survivors, two age-related dimensions can be distinguished: current age, the life stage in which dietary behavior is enacted, and age at diagnosis, the life stage in which the cancer experience first occurred. While current age is routinely accounted for in dietary studies, survivors are less often distinguished by their age at diagnosis—even though a diagnosis earlier in life tends to occur during a life stage characterized by competing demands such as employment and child-rearing, which may shape whether healthier diets are adopted and sustained with age.

Although health-related behaviors, including diet, are known to vary with time since diagnosis [12], prior studies have largely treated cancer survivors as a single group and have rarely distinguished survivors by their age at diagnosis. As a result, whether and how dietary quality differs by age at diagnosis—and how any such difference evolves across a survivor’s current age—remains unclear at the population level. Addressing this question requires a large, nationally representative sample in which survivors diagnosed at different life stages can be compared with the general population across the full adult age range.

One way to organize these two dimensions is the life course perspective on health, which holds that health behaviors and outcomes are shaped both by the timing of significant events and by the accumulation of advantage or disadvantage over time [13]. In this framework, age at diagnosis reflects the timing of a major health event and current age the period over which post-diagnosis behaviors accumulate, offering one plausible basis for expecting that survivors’ dietary quality may depend jointly on when the cancer was diagnosed and how much of the life course has since elapsed.

The Diet Quality Index-International (DQI-I) is a validated tool designed to evaluate overall dietary quality across diverse populations, accounting for both nutritional adequacy and chronic disease risk factors [14]. In this study, we used nationally representative data from the KNHANES, together with a survey-weighted analytic approach, to compare dietary quality between cancer survivors and the general population using the DQI-I, and—uniquely—to examine how dietary quality among survivors differs jointly by age at diagnosis and current age. By leveraging a large population-based sample, this study extends prior work limited to single dimensions of age or to smaller clinical samples, and provides the first nationally representative characterization of how the survivor dietary advantage is distributed across life stages.

## 2. Materials and Methods

### 2.1. Study Participants

This study utilized data from the eighth Korea National Health and Nutrition Examination Survey (KNHANES VIII), conducted between 2019 and 2021. KNHANES is a nationally representative survey conducted by the Korea Disease Control and Prevention Agency, designed to provide reliable data for establishing and evaluating public health policies aimed at improving the health and nutrition of the Korean population. It uses a stratified, multistage clustered probability sampling design, and sampling weights accounting for unequal selection probabilities, non-response, and post-stratification to the Korean population were applied in all analyses [15].

Out of a total of 22,559 participants, 13,602 individuals who answered “followed their diet as usual on the previous day” were included in the analysis. We excluded 3517 individuals younger than the age of 30, 467 individuals with missing data on cancer diagnosis, and 912 individuals with missing data on the variables analyzed, resulting in a final study sample of 8706 participants. This sample represents a weighted population of 23,659,361 individuals.

Among them, 641 were classified as the cancer survivor group and 8065 as the control group. In this study, cancer survivors were defined as adults aged 30 years or older who responded “yes” to having ever been diagnosed with cancer.

This study was deemed exempt from review by the Institutional Review Board of Seoul National University Bundang Hospital (Approval No. X-2507-985-904) under 45 CFR 46.101(b).

### 2.2. Measurements

Data from self-reported questionnaires were used, including variables such as age, gender, marital status, educational level, monthly income, residential area, smoking status, alcohol consumption, and presence of comorbid chronic diseases.

Body mass index (BMI) was calculated using height (cm) and weight as weight (kg) divided by height squared (m^2^). Marital status was categorized as married or unmarried. Educational level was classified into three groups: less than elementary school graduation, middle/high school graduation, and university graduation or higher.

Monthly income was grouped into three categories: less than 2 million KRW, 2–4 million KRW, and more than 4 million KRW. Residential area was classified as urban or rural. Smoking status was categorized as current smoker, former smoker, or non-smoker. Alcohol consumption was classified as current drinker, former drinker, or non-drinker. Comorbid chronic diseases included hypertension, dyslipidemia, diabetes, stroke, cardiovascular disease, chronic kidney disease, and osteoarthritis, and were classified based on the presence of at least one of these conditions.

### 2.3. Evaluation of Dietary Quality

Dietary assessments were conducted using a 24 h dietary recall method, which was administered by trained dietitians. The analysis and evaluation of diet quality were performed using the DQI-I. The DQI-I consists of four components: variety (20 points), adequacy (40 points), moderation (30 points), and overall balance (10 points), with a total score of 100 points. Higher scores indicate better diet quality [14].

The four components are defined as follows. Variety quantifies dietary diversity through overall food group variety (meat/poultry/fish/eggs, dairy/legumes, grains, fruits, and vegetables; up to 15 points) and within-group variety of protein sources (up to 5 points). Adequacy evaluates the intake of beneficial dietary elements—vegetables, fruits, grains, fiber, protein, iron, calcium, and vitamin C—each scored from 0 to 5 points. Moderation evaluates the intake of elements recommended for limitation, including total fat, saturated fat, cholesterol, sodium, and empty-calorie foods, each scored from 0 to 6 points. Overall balance evaluates dietary proportionality based on the macronutrient energy ratio (carbohydrate, protein, and fat; up to 6 points) and the fatty acid ratio (polyunsaturated, monounsaturated, and saturated fatty acids; up to 4 points). Component and total scores were derived using the original DQI-I scoring algorithm [14].

For the adequacy component, the evaluation of the percentage intake of each nutrient was based on the “Dietary Reference Intakes for Koreans” published by the Korean Nutrition Society in 2020 [16]. Empty-calorie foods were defined as high-calorie foods composed mostly of carbohydrates and fats, with little to no essential nutrients such as vitamins and amino acids, such as ice cream and cookies [17].

### 2.4. Statistical Analysis

The general characteristics of the study population were presented as survey-weighted means with standard errors for continuous variables and as unweighted frequencies with survey-weighted percentages for categorical variables. Between-group comparisons of sociodemographic characteristics were based on survey-weighted univariate regression, accounting for the complex sampling design.

To examine factors associated with DQI-I score, survey-weighted univariate linear regression analyses were conducted. Variables that showed significant differences between the two groups—including age, sex, BMI, marital status, educational attainment, monthly income, residential area, smoking status, alcohol consumption, and presence of chronic diseases—were included as covariates in survey-weighted multiple linear regression models to assess differences in DQI-I scores between cancer survivors and controls. Full outputs of all multivariable regression models, including models stratified by age at diagnosis and current age, are provided in Supplementary Table S1.

To investigate whether the association between age and DQI-I score differed by age at cancer diagnosis, stratified analyses were performed. A diagnostic age cutoff of 50 years was selected, corresponding to the widely accepted clinical definition of early-onset cancer, which refers to cancers diagnosed before the age of 50 and is increasingly recognized as a distinct entity with different etiologic and biological characteristics [18]. This threshold is further supported by evidence that social and physical activity levels tend to peak and subsequently decline around this age [19,20], which may also influence dietary behaviors.

**Figure 1:**
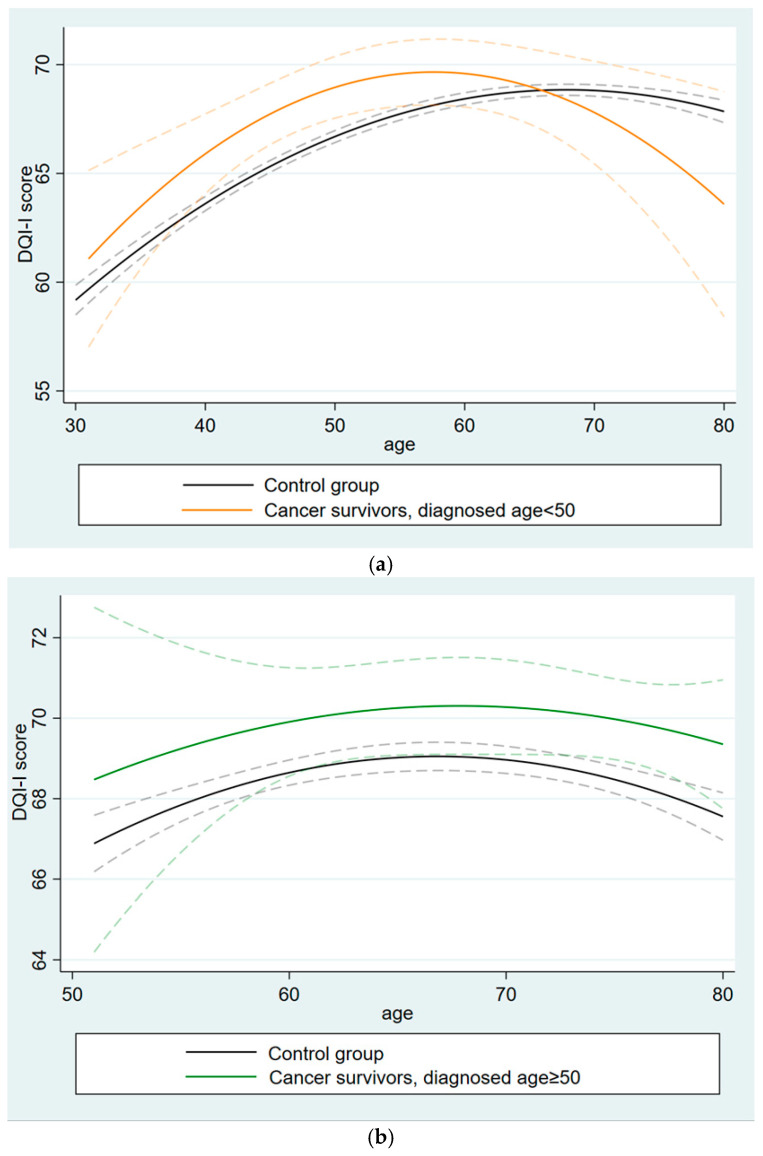
Association between current age and diet quality (DQI-I score) among cancer survivors vs. controls, stratified by survivors’ age at cancer diagnosis. (**a**) Association between age and DQI-I scores among the control group and cancer survivors diagnosed before age 50. (**b**) Association between age and DQI-I scores among survivors diagnosed at age 50 or older and the control group. Solid lines show survey-weighted quadratic regression fits; dashed lines show 95% confidence intervals, illustrating the uncertainty around the fitted mean DQI-I trajectory at each age.

In addition, a quadratic term for age (age^2^) was included in the model to assess potential nonlinear associations, and model fit was evaluated by comparing R-squared values. Model comparison showed that adding a quadratic age term improved model fit in controls and in survivors diagnosed before age 50, with significant negative coefficients for age squared, whereas little improvement was observed among survivors diagnosed at or after age 50 (Supplementary Table S2). Therefore, quadratic age terms were used to capture potential nonlinear age-related patterns, particularly in the control and younger-onset survivor groups. Accordingly, survey-weighted quadratic regression models were applied to visualize and quantify the relationship between age and DQI-I score across strata defined by age at diagnosis. For the stratified subgroup analysis of current age, a cutoff at 65 years was selected based on observed divergence in DQI-I trends among those diagnosed before age 50 (Figure 1a). Because this cutoff was partly informed by the fitted curves, sensitivity analyses using alternative current-age cutoffs of 60 and 70 years were additionally performed.

Group differences in mean DQI-I scores across controls and cancer survivor subgroups were evaluated using survey-weighted linear regression. Overall differences across the three groups were assessed using a design-based F-test (the survey-weighted analog of one-way ANOVA), and pairwise differences between each survivor subgroup and controls were obtained from the same models.

All statistical analyses were conducted using STATA version 16.0 (StataCorp, College Station, TX, USA), and a *p*-value < 0.05 was considered statistically significant.

## 3. Results

### 3.1. Study Population Characteristics

The mean age was 52.5 ± 11.0 years in the group of cancer survivors diagnosed before the age of 50, 70.1 ± 7.7 years in those diagnosed at or after the age of 50, and 57.6 ± 14.1 years in the control group. The general characteristics of each group are summarized in Table 1. The survivor group comprised diverse cancer types, most commonly thyroid (*n* = 122), gastric (*n* = 107), colorectal (*n* = 91), and breast (*n* = 92) cancers (Supplementary Table S3).

### 3.2. Diet Quality Assessment Based on DQI-I Scores

The mean total DQI-I score for the overall study population was 66.26 ± 0.16. Cancer survivors had significantly higher total scores than the control group (69.08 ± 0.41 vs. 66.07 ± 0.16, *p* < 0.001). A detailed comparison of DQI-I total and component scores between cancer survivors and the control group is presented in Table 2. At the component level, cancer survivors scored significantly higher than controls in variety, adequacy, and moderation (all *p* < 0.05). Overall balance was the only component with a lower mean score among survivors; however, this difference was not statistically significant (2.90 ± 0.14 vs. 3.08 ± 0.04, *p* = 0.223), indicating no meaningful between-group difference in this component.

When cancer survivors were further stratified by age at diagnosis, the mean total DQI-I score was 67.92 ± 0.72 among those diagnosed before age 50 and 69.83 ± 0.46 among those diagnosed at or after age 50, compared to 66.07 ± 0.16 in the control group (*p* < 0.001). Pairwise comparisons showed that survivors diagnosed before age 50 had a significantly higher DQI-I score than controls (*p* = 0.012), as did those diagnosed at ≥50 years (*p* < 0.001). A detailed comparison of total and component DQI-I scores according to age at cancer diagnosis is presented in Table 3. At the component level, the differences that reached statistical significance were consistently in favor of survivors: variety was higher in those diagnosed before age 50 (*p* = 0.005), and adequacy and moderation were higher in those diagnosed at or after age 50 (both *p* < 0.001). Overall balance in the later-onset group (2.79 ± 0.15 vs. 3.08 ± 0.04) showed a lower mean than controls, but this difference was not statistically significant (*p* = 0.069).

### 3.3. Subgroup Analysis by Age at Diagnosis and Current Age

To explore potential differences in dietary quality by current age following cancer diagnosis, a subgroup analysis was conducted based on age at diagnosis and current age. Survivors diagnosed before age 50 were further divided by current age into those under 65 years and those aged 65 years or older, based on the observation that their mean DQI-I scores crossed over those of the control group around age 65.

In survivors diagnosed before age 50, diet quality was initially higher than that of the general population but was progressively lower across older age groups with increasing age, crossing below control levels around age 65 (Figure 1a). This crossover was supported by survey-weighted linear regression: those currently under 65 (*n* = 174) had significantly higher DQI-I scores than controls (mean difference = 3.02; 95% CI: 1.44–4.60; *p* < 0.001), whereas those aged 65 or older (*n* = 29) had significantly lower scores (mean difference = −3.18; 95% CI: −6.16 to −0.20; *p* = 0.036) (Table 4). Confidence bands widen at older ages among survivors diagnosed before age 50, reflecting fewer observations in this region. To assess whether the findings were dependent on the current-age cutoff of 65 years, sensitivity analyses were performed using alternative cutoffs of 60 and 70 years. The results showed a generally similar age-dependent pattern: survivors diagnosed before age 50 had higher DQI-I scores than controls in the younger current-age strata, whereas this difference was attenuated or reversed in the older strata (Supplementary Table S4). In survey-weighted multivariable regression models, survivors diagnosed before age 50 had significantly higher DQI-I scores than controls among those currently under 65 years (β = 1.57; 95% CI: 0.09–3.04; *p* = 0.037), whereas they had significantly lower scores among those aged 65 years or older (β = −2.89; 95% CI: −5.56 to −0.22; *p* = 0.034) (Supplementary Table S1).

In contrast, survivors diagnosed at or after age 50 (*n* = 438; *n* = 119 aged < 65 and *n* = 319 aged ≥ 65) consistently showed higher diet quality scores than the general population across all age groups (Figure 1b), with a significant overall difference (mean difference = 3.76; 95% CI: 2.83–4.68; *p* < 0.001) (Table 4).

## 4. Discussion

This study investigated dietary habits among cancer survivors compared to the general population using the Diet Quality Index-International (DQI-I), with a particular focus on the role of age at cancer diagnosis in dietary quality across age groups. The findings offer insights into how survivorship experiences, especially age at diagnosis, may be associated with dietary quality.

Dietary habits among cancer survivors have been a focus of many previous studies, often emphasizing the association between specific dietary components and long-term prognosis in cancers such as breast, prostate, and colorectal cancer. For instance, low-fat dietary patterns have been linked to reduced recurrence and mortality in breast cancer survivors [21] and higher fruit and vegetable intake has been associated with improved outcomes in prostate and colorectal cancer [22,23]. These findings have contributed to the development of evidence-based dietary guidelines for cancer survivors, including guidelines by the American Cancer Society [24]. These guidelines consistently emphasize the consumption of a variety of vegetables, fruits, whole grains, and legumes, while recommending limitations on added sugars, saturated fats, processed foods, and red or processed meats to maintain adequate body weight [25]. Despite the availability of these guidelines, studies consistently report low adherence among cancer survivors. Several studies show that a large proportion of survivors did not meet recommendations for key dietary components [7], highlighting the challenges of long-term dietary maintenance in this population.

In contrast to these findings, our study showed that cancer survivors had significantly higher DQI-I scores than the general population. Across all DQI-I components that differed significantly from controls, cancer survivors consistently scored higher, consistent with their higher overall diet quality. This discrepancy may be partly explained by differences in cultural dietary patterns, particularly the traditional Korean diet, which is generally rich in vegetables, fermented foods, and whole grains, and relatively low in fat compared to Western diets [26]. Since the mean age of cancer survivors in our sample was significantly higher than that of the control group, and given that older adults in Korea are more likely to adhere to traditional dietary practices [27], age itself may have acted as a confounding factor in the observed association.

Our analysis further revealed that this pattern of higher diet quality among survivors varied according to age at diagnosis. Notably, survivors diagnosed before age 50 initially demonstrated higher DQI-I scores compared to controls, but this advantage was attenuated in older age groups, becoming lower than that of the general population after age 65, as shown in Figure 1a. In contrast, those diagnosed at age 50 or older showed consistently higher diet quality scores across all age groups, as shown in Figure 1b. These results suggest that while younger-onset cancer survivors may initially adopt healthy dietary behaviors, they show lower dietary quality at older ages.

Several factors may contribute to this pattern, which can be viewed through a life course perspective on health [13]. Younger survivors may have greater initial motivation for dietary change after diagnosis—driven by heightened health consciousness and fear of recurrence [28]—but may face challenges maintaining such behaviors over a longer survivorship trajectory due to life demands such as career responsibilities or parenting [29,30]; in life course terms, an early dietary advantage may erode with the cumulation of these competing demands over time. Older survivors, in turn, may have more established dietary routines and more consistent healthcare contact [31], which may help sustain a more stable advantage across age. As these factors were not measured in the present study, these interpretations remain speculative and should be confirmed in future research.

This study has several strengths. First, it used nationally representative data of the Korean population from KNHANES, which enhances the generalizability of our findings to the broader Korean adult survivor population. Second, the use of DQI-I allowed for a comprehensive evaluation of diet quality across multiple dimensions. Additionally, the application of survey-weighted regression models and stratified quadratic analysis enabled a nuanced assessment of nonlinear patterns in diet quality across age. Furthermore, this study offers a novel perspective by examining how age at cancer diagnosis may be associated with dietary quality.

Nevertheless, several limitations should be acknowledged. First, the cross-sectional nature of the study precludes causal inference. We compared different individuals at different ages, rather than following the same survivors over time. Accordingly, the observed lower dietary scores among older survivors diagnosed at a younger age should be interpreted as a cohort difference, and confirmation with longitudinal data would be essential. Second, the current-age cutoff of 65 years was selected after visual inspection of the fitted curves and may therefore be subject to data-driven subgroup definition or overfitting. Although 65 years is commonly used to define older adults and sensitivity analyses using alternative cutoffs of 60 and 70 years showed generally similar age-dependent patterns, the exact cutoff should not be interpreted as definitive. Therefore, these subgroup findings should be regarded as exploratory and interpreted with caution. Third, survivor bias should be acknowledged. As the included survivors are necessarily those who survived after diagnosis, individuals with healthier lifestyles may be overrepresented, potentially limiting generalizability. Nevertheless, this is unlikely to be the primary explanation for our findings, as the longest-surviving subgroup (diagnosed before age 50, currently aged ≥ 65)—those most prone to healthy-survivor selection—showed lower rather than higher diet quality than the general population. Fourth, dietary intake was assessed using a single 24 h recall, which reflects intake on the preceding day rather than habitual or long-term dietary behavior and is subject to recall bias. The DQI-I scores should therefore be interpreted as a single-day estimate of diet quality rather than sustained adherence. Nevertheless, this method is widely used in national surveys and provides standardized, population-level estimates of diet quality. Fifth, we were unable to account for time since diagnosis. In survivorship research, time since diagnosis can influence lifestyle behaviors, but in cross-sectional data, it is closely tied to current age and age at diagnosis. As a result, differences observed by age at diagnosis cannot be fully separated from differences in survivorship duration. Future longitudinal studies incorporating time since diagnosis are warranted to disentangle these dimensions. Finally, the survivor group was heterogeneous in cancer type, which differs in treatment and dietary implications; for example, gastrointestinal cancers may entail post-surgical dietary restrictions and altered nutrient absorption [32], whereas thyroid and breast cancers impose fewer constraints. Because the DQI-I reflects overall rather than cancer-specific diet quality and subgroup sizes were too small for type-specific analyses, pooling may obscure type-specific patterns, which warrant dedicated future study.

Despite these limitations, this study contributes valuable knowledge regarding the heterogeneity of dietary behavior among cancer survivors and suggests opportunities for more personalized survivorship care. As cancer survivors are at higher risk of developing second primary cancers and other chronic diseases compared to the general population [33,34], there is a need for ongoing dietary assessment and education regardless of survival duration. Future research should explore the mechanisms underlying the lower diet quality observed among older survivors diagnosed at a younger age and identify strategies for interventions tailored to age and time since diagnosis to support sustainable dietary improvements across the survivorship continuum.

Our findings highlight that age at diagnosis may be associated with dietary quality among cancer survivors. Younger survivors may require ongoing, structured support to sustain initial behavioral changes, whereas older survivors may benefit more from age-tailored dietary interventions. Such support could include periodic dietary assessment, individualized counseling by clinical dietitians, and repeated nutrition education integrated into routine survivorship follow-up, rather than guidance delivered only around the time of diagnosis.

## 5. Conclusions

Cancer survivors exhibited overall higher dietary quality than the general population; however, this advantage was not consistent across all groups. Notably, survivors diagnosed at a younger age showed lower dietary quality in older age groups, whereas those diagnosed later showed consistently favorable dietary quality. These findings highlight that dietary quality may vary according to age at cancer diagnosis and current age among cancer survivors. Given the elevated risk of secondary health outcomes in this population, sustained and tailored nutritional interventions—particularly for younger-onset survivors—are warranted to support long-term health and improve survivorship outcomes.

## Figures and Tables

**Table 1:** General characteristics of adults aged ≥ 30 years by cancer survivorship status, KNHANES VIII (2019–2021).

Characteristic	Total (*n* = 8706)	Controls (*n* = 8065)	Cancer Survivors		*p*-Value
			Age at Diagnosis		
			<50 (*n* = 203)	≥50 (*n* = 438)	
Age	58.1 ± 14.1	57.6 ± 14.1	52.5 ± 11.0	70.1 ± 7.7	<0.001
Sex					
Male	3812 (43.8)	3538 (43.9)	42 (20.7)	232 (53.0)	0.001
Female	4894 (56.2)	4527 (56.1)	161 (79.3)	206 (47.0)	
BMI	24.0 ± 3.4	24.0 ± 3.4	23.7 ± 3.5	23.5 ± 3.3	0.006
Marital status					<0.001
Married	8096 (93.0)	7474 (92.7)	190 (93.6)	432 (98.6)	
Unmarried	610 (7.0)	610 (7.3)	13 (6.4)	6 (1.4)	
Education					<0.001
≥College	2994 (33.8)	2785 (34.5)	98 (48.3)	61 (13.9)	
Middle/high school	3646 (41.9)	3362 (41.7)	76 (37.4)	208 (47.5)	
≤Elementary school	2116 (24.3)	1918 (23.8)	29 (14.3)	169 (38.6)	
Monthly income					<0.001
≥4 million	3740 (43.0)	3519 (43.6)	119 (58.6)	102 (23.3)	
2–4 million	2163 (24.8)	2012 (25.0)	49 (24.1)	102 (23.3)	
≤2 million	2803 (32.2)	2534 (31.4)	35 (17.2)	234 (53.4)	
Residential area					0.232
Urban	6711 (77.1)	6231 (77.3)	165 (81.3)	315 (71.9)	
Rural	1995 (22.9)	1834 (22.7)	38 (18.7)	123 (28.1)	
Smoking					<0.001
Current smoker	1370 (15.7)	1312 (16.3)	16 (7.9)	42 (9.6)	
Ex-smoker	2114 (24.3)	1921 (23.8)	34 (16.8)	159 (36.3)	
Never smoker	5222 (60.0)	4832 (59.9)	153 (75.3)	237 (54.1)	
Alcohol					<0.001
Current drinker	5559 (63.9)	5249 (65.1)	135 (66.5)	175 (40.0)	
Ex-drinker	1919 (22.0)	1703 (21.1)	46 (22.7)	170 (38.8)	
Never drinker	1228 (14.1)	1113 (13.8)	22 (10.8)	93 (21.2)	
Chronic disease					<0.001
Absent	6224 (71.5)	5794 (71.8)	166 (81.8)	264 (60.3)	
Present	2482 (28.5)	2271 (28.2)	37 (18.2)	174 (39.7)	

Values are presented as means ± standard deviations or numbers (%). *p*-values were calculated by survey-weighted univariate logistic regressions for categorical variables and survey-weighted univariate linear regressions for continuous variables. BMI, body mass index.

**Table 2:** Comparison of DQI-I component scores between cancer survivors and the control group.

DQI Components	Total (*n* = 8706)	Controls (*n* = 8065)	Cancer Survivors (*n* = 641)	*p* Value *
DQI-I overall score(100)	66.26 ± 0.16	66.07 ± 0.16	69.08 ± 0.41	<0.001
Variety (20)	16.44 ± 0.05	16.41 ± 0.05	16.78 ± 0.12	0.002
Adequacy(40)	27.84 ± 0.09	27.76 ± 0.09	28.93 ± 0.27	<0.001
Moderation (30)	18.92 ± 0.11	18.82 ± 0.11	20.48 ± 0.31	<0.001
Overall balance(10)	3.06 ± 0.04	3.08 ± 0.04	2.90 ± 0.14	0.223

Values are presented as survey-weighted means ± standard errors; reported sample sizes (*n*) are unweighted. * *p*-values were derived from survey-weighted linear regression. DQI-I, Diet Quality Index-International.

**Table 3:** Comparison of DQI-I component scores by age at cancer diagnosis and the control group.

DQI Components	Total (*n* = 8706)	Controls (*n* = 8065)	Cancer Survivors		*p* Value * (Overall)	Pairwise *p* Value ^†^ (Control vs. Age < 50)	Pairwise *p* Value ^†^ (Control vs. Age ≥ 50)
			Age at Diagnosis				
			<50 (*n* = 203)	≥50 (*n* = 438)			
DQI-I overall score (100)	66.26 ± 0.16	66.07 ± 0.16	67.92 ± 0.72	69.83 ± 0.46	<0.001	0.012	<0.001
Variety (20)	16.44 ± 0.05	16.41 ± 0.05	16.96 ± 0.19	16.67 ± 0.15	0.005	0.005	0.096
Adequacy (40)	27.84 ± 0.09	27.76 ± 0.09	28.54 ± 0.42	29.18 ± 0.31	<0.001	0.070	<0.001
Moderation (30)	18.92 ± 0.11	18.82 ± 0.11	19.36 ± 0.58	21.19 ± 0.34	<0.001	0.357	<0.001
Overall balance (10)	3.06 ± 0.04	3.08 ± 0.04	3.07 ± 0.26	2.79 ± 0.15	0.189	0.979	0.069

Values are presented as survey-weighted means ± standard errors; reported sample sizes (*n*) are unweighted. DQI-I, Diet Quality Index-International. * Overall *p*-values were derived from survey-weighted linear regression, using a design-based F-test (the survey-weighted analog of one-way ANOVA) across the three groups. ^†^ Pairwise *p*-values represent comparisons of each survivor subgroup with controls from the same survey-weighted regression models.

**Table 4:** Survey-weighted mean differences in DQI-I score relative to controls among cancer survivors, by age at diagnosis and current age.

Age at Diagnosis	Current Age	*n*	Control DQI-I (Mean ± SE)	Cancer Survivors DQI-I (Mean ± SE)	Mean Difference vs. Controls	95% CI	*p* Value
Before age 50	<65	174	65.19 ± 0.19	68.21 ± 0.79	+3.02	1.44 to 4.60	<0.001
Before age 50	≥65	29	68.74 ± 0.21	65.55 ± 1.53	−3.18	−6.16 to −0.20	0.036
At or after age 50	all	438	66.07 ± 0.16	69.83 ± 0.46	+3.76	2.83 to 4.68	<0.001

Values represent survey-weighted mean differences in DQI-I score relative to controls, derived from survey-weighted linear regression. *n* values are unweighted. DQI-I, Diet Quality Index-International. CI, Confidence Interval.

## Data Availability

The data presented in this study are available through the Korea National Health and Nutrition Examination Survey (KNHANES) official website (https://knhanes.kdca.go.kr/knhanes (accessed on 14 October 2024)) upon request and approval by the Korea Disease Control and Prevention Agency.

## Supplementary Materials

The following supporting information can be downloaded at https://www.mdpi.com/article/10.3390/nu18132172/s1: Table S1: Survey-weighted multivariable linear regression models for DQI-I score (full model outputs); Table S2: Comparison of linear and quadratic age models for DQI-I score; Table S3: Number of cancer survivors by cancer type and age at cancer diagnosis, KNHANES VIII (2019–2021); Table S4: Sensitivity analyses using alternative current-age cutoffs among cancer survivors diagnosed before age 50.

## Abbreviations

The following abbreviations are used in this manuscript:

ANOVA	Analysis of variance
BMI	Body mass index
CI	Confidence interval
DQI-I	Diet Quality Index-International
KDCA	Korea Disease Control and Prevention Agency
KNHANES	Korea National Health and Nutrition Examination Survey
