# Supplementary material for: Dietary Quality Changes Among Cancer Survivors Compared with Age at Cancer Diagnosis: Using the Korean National Health and Nutrition Examination Surveys (KNHANES 2019–2021)
Source: Nutrients. 2026 Jul 4;18(13):2172. doi: 10.3390/nu18132172 (PMC13363523; doi:10.3390/nu18132172)
Supplement: Supplementary file 1 [file nutrients-18-02172-s001.zip › nutrients-4380939-supplementary.pdf]

Supplementary Table S1. Survey-weighted multivariable linear regression models for DQI-I score (full model outputs).

	Model 1<br>Cancer survivor vs. control			Model 2<br>Age at diagnosis vs. control			Model 3<br>Diagnosed <50 vs. control (age <65)			Model 4<br>Diagnosed <50 vs. control (age ≥65)		
**Variable**	**β**	**95% CI**	**p**	**β**	**95% CI**	**p**	**β**	**95% CI**	**p**	**β**	**95% CI**	**p**
**Main exposure**												
Cancer survivor (ref: control)	0.95	0.17, 1.74	0.017	—	—	—	—	—	—	—	—	—
Diagnosed <50 (ref: control)	—	—	—	1.03	−0.30, 2.37	0.130	—	—	—	—	—	—
Diagnosed ≥50 (ref: control)	—	—	—	0.90	0.01, 1.79	0.048	—	—	—	—	—	—
Diagnosed <50 (ref: control)	—	—	—	—	—	—	1.57	0.09, 3.04	0.037	−2.89	−5.56, −0.22	0.034
**Covariates**												
Age (years)	0.80	0.65, 0.95	<0.001	0.80	0.65, 0.95	<0.001	0.56	0.22, 0.90	0.001	2.97	0.49, 5.46	0.019
Age²	−0.005	−0.006, −0.004	<0.001	−0.005	−0.006, −0.004	<0.001	−0.003	−0.006, 0.001	0.155	−0.021	−0.038, −0.003	0.018
**Sex (ref: male)**												
Female	0.68	0.04, 1.33	0.038	0.68	0.04, 1.33	0.039	0.88	0.10, 1.65	0.028	0.02	−1.02, 1.06	0.969
BMI (kg/m²)	−0.05	−0.12, 0.02	0.161	−0.05	−0.12, 0.02	0.160	−0.05	−0.14, 0.03	0.245	0.004	−0.11, 0.12	0.946
**Marital status (ref: married/cohabiting)**												
Other	1.25	0.26, 2.25	0.014	1.25	0.26, 2.25	0.014	1.19	0.15, 2.23	0.025	−2.11	−6.51, 2.28	0.346
**Education (ref: ≥college)**												
Middle/high school	−0.83	−1.40, −0.26	0.004	−0.83	−1.40, −0.26	0.004	−0.88	−1.51, −0.24	0.007	−0.94	−2.23, 0.34	0.149
≤Elementary school	−3.21	−3.98, −2.45	<0.001	−3.21	−3.98, −2.45	<0.001	−3.20	−4.48, −1.91	<0.001	−3.33	−4.63, −2.03	<0.001
**Monthly income (ref: >4 million KRW)**												
2–4 million KRW	−0.46	−1.08, 0.17	0.152	−0.46	−1.08, 0.17	0.152	−0.83	−1.53, −0.13	0.020	0.91	−0.39, 2.21	0.170
≤2 million KRW	−1.96	−2.68, −1.23	<0.001	−1.95	−2.68, −1.23	<0.001	−1.76	−2.70, −0.82	<0.001	−1.23	−2.41, −0.05	0.042
**Residential area (ref: urban)**												
Rural	−0.19	−0.79, 0.41	0.528	−0.19	−0.79, 0.41	0.528	0.25	−0.51, 1.02	0.515	−0.92	−1.86, 0.02	0.056
**Smoking (ref: current smoker)**												
Ex-smoker	3.33	2.63, 4.03	<0.001	3.33	2.63, 4.03	<0.001	3.16	2.34, 3.98	<0.001	3.50	2.21, 4.80	<0.001
Never smoker	4.46	3.63, 5.29	<0.001	4.46	3.63, 5.29	<0.001	4.26	3.28, 5.24	<0.001	4.98	3.49, 6.47	<0.001
**Alcohol (ref: current drinker)**												
Ex-drinker	1.49	1.00, 1.97	<0.001	1.49	1.00, 1.98	<0.001	1.61	0.95, 2.26	<0.001	0.86	0.05, 1.68	0.038
Never drinker	1.09	0.38, 1.81	0.003	1.09	0.38, 1.81	0.003	1.76	0.74, 2.78	0.001	0.53	−0.45, 1.51	0.289
**Chronic disease (ref: absent)**												
Present	−0.29	−0.85, 0.26	0.301	−0.29	−0.85, 0.26	0.301	−0.70	−1.58, 0.18	0.121	0.02	−0.73, 0.78	0.953
Constant	37.10	32.56, 41.64	<0.001	37.11	32.56, 41.67	<0.001	42.67	34.44, 50.89	<0.001	−40.28	−130.26, 49.71	0.380
**R²**	0.1532			0.1532			0.1483			0.0787		
**N**	8,706			8,706			5,391			2,877		

Note. Model 1: all cancer survivors vs. controls. Model 2: survivors diagnosed before age 50 and those diagnosed at or after age 50 simultaneously vs. controls (single regression model, controls as reference). Model 3: survivors diagnosed before age 50 vs. controls, restricted to current age <65 years. Model 4: survivors diagnosed before age 50 vs. controls, restricted to current age ≥65 years. All models were adjusted for age, age squared, sex, body mass index, marital status, education, monthly income, residential area, smoking status, alcohol consumption, and chronic disease status. Coefficients represent covariate-adjusted differences and therefore differ in magnitude from the unadjusted survey-weighted mean differences reported in the main text (Section 3.3); both analyses showed consistent direction and statistical significance. β,

regression coefficient; CI, confidence interval; DQI-I, Diet Quality Index-International; KRW, Korean Won.

Supplementary Table S2. Comparison of linear and quadratic age models for DQI-I score.

Group	β for age²	p-value for age²	Linear R²	Quadratic R²	ΔR²
Controls	−0.0059	<0.001	0.0724	0.0870	0.0146
Survivors diagnosed before age 50	−0.0156	0.0008	0.0252	0.0970	0.0718
Survivors diagnosed at or after age 50	−0.0009	0.916	0.0002	0.0002	0.0000

Note. Values were derived from survey-weighted regression models within each group. Linear models included current age as the independent variable, and quadratic models included both current age and age squared. The p-value for age² was obtained from the adjusted Wald test. ΔR² was calculated as quadratic R² minus linear R². DQI-I, Diet Quality Index-International.

Supplementary Table S3. Number of cancer survivors by cancer type and age at cancer diagnosis, KNHANES VIII (2019–2021).

Cancer type	Diagnosed <50, n	Diagnosed ≥50, n	Total, n
Gastric	18	89	107
Liver	0	11	11
Colorectal	21	70	91
Breast	34	58	92
Cervical	21	22	43
Lung	4	30	34
Thyroid	75	47	122
Other	36	135	171

Note. Values are numbers of cancer survivors classified by age at cancer diagnosis (before age 50 vs. age 50 or older). Patients diagnosed with more than one primary cancer are counted under each applicable cancer type; therefore, column totals exceed the number of unique survivors. For each cancer type, the Total column reflects all survivors with that diagnosis, and the two age-at-diagnosis subgroups may not sum to the total when age at diagnosis was not reported. KNHANES, Korea National Health and Nutrition Examination Survey.

Supplementary Table S4. Sensitivity analyses using alternative current-age cutoffs among cancer survivors diagnosed before age 50.

Current-age cutoff	Age stratum	Controls, mean ± SE	Survivors diagnosed before age 50, mean ± SE	Mean difference	95% CI	p-value
60 years	<60 years	64.65 ± 0.20	67.73 ± 0.84	3.08	1.41 to 4.74	<0.001
60 years	≥60 years	68.77 ± 0.19	68.79 ± 1.37	0.03	−2.66 to 2.71	0.985
65 years	<65 years	65.19 ± 0.19	68.21 ± 0.79	3.02	1.44 to 4.60	<0.001
65 years	≥65 years	68.74 ± 0.21	65.55 ± 1.53	−3.18	−6.16 to −0.20	0.036
70 years	<70 years	65.54 ± 0.18	68.18 ± 0.76	2.64	1.11 to 4.17	<0.001
70 years	≥70 years	68.75 ± 0.24	64.27 ± 1.78	−4.47	−7.95 to −1.00	0.012

Note. Values are survey-weighted means ± standard errors. Mean differences indicate DQI-I scores in cancer survivors diagnosed before age 50 minus those in controls within each current-age stratum. P-values were obtained from survey-weighted linear regression models including only group status, corresponding to survey-weighted mean comparisons. DQI-I, Diet Quality Index-International; CI, confidence interval; SE, standard error.
